# Supplementary figures and images for: MiR-150-5p Overexpression in Triple-Negative Breast Cancer Contributes to the In Vitro Aggressiveness of This Breast Cancer Subtype
Source: Cancers (Basel). 2022 Apr 26;14(9):2156. doi: 10.3390/cancers14092156 (PMC9104497; doi:10.3390/cancers14092156)

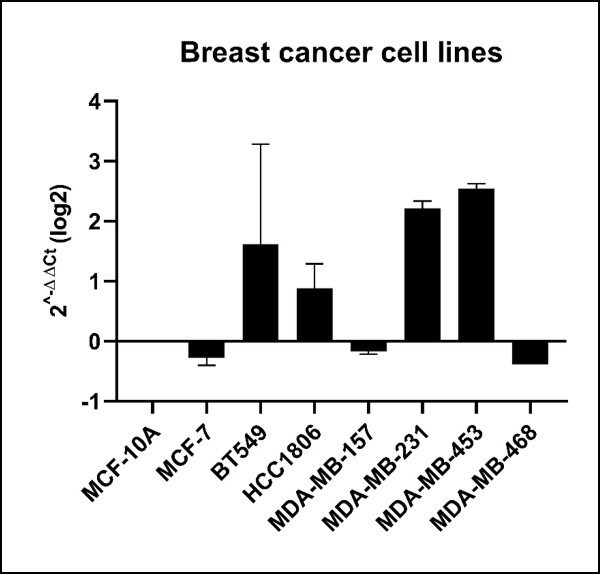

Supplement: Supplementary file 1 [file cancers-14-02156-s001.zip › Figure S1.jpg]

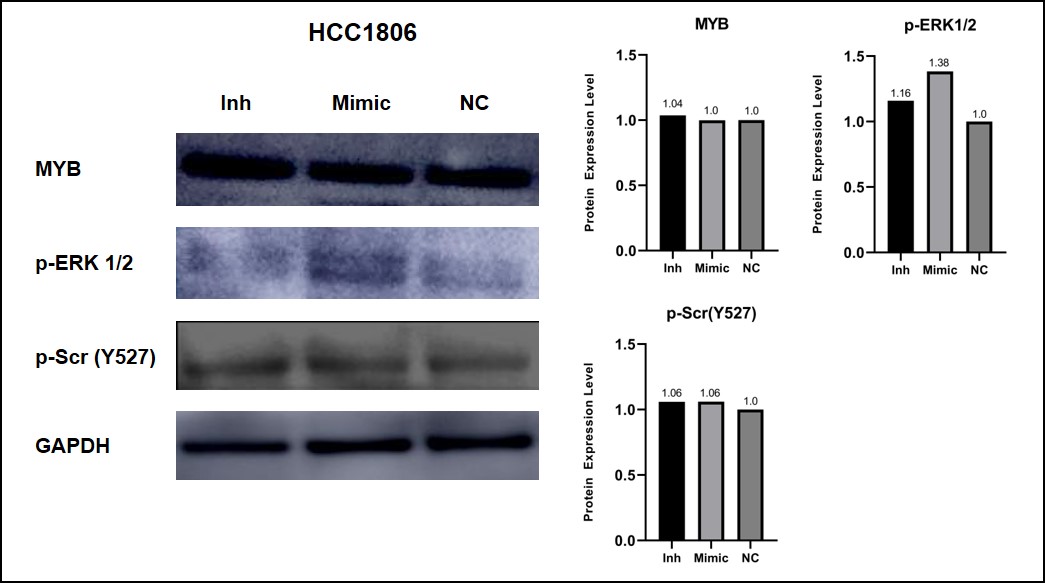

Supplement: Supplementary file 1 [file cancers-14-02156-s001.zip › Figure S2.jpg]
